# Erratum to: Integrated printed BDNF/collagen/chitosan scaffolds with low temperature extrusion 3D printer accelerated neural regeneration after spinal cord injury

**DOI:** 10.1093/rb/rbab063

**Published:** 2022-04-26

**Authors:** Xiao-Yin Liu, Chong Chen, Hai-Huan Xu, Yu-sheng Zhang, Lin Zhong, Nan Hu, Xiao-Li Jia, You-Wei Wang, Kun-Hong Zhong, Chang Liu, Xu Zhu, Dong Ming, Xiao-Hong Li

**Affiliations:** 1 Tianjin Key Laboratory of Brain Science and Neural Engineering, Academy of Medical Engineering and Translational Medicine, Tianjin University, Tianjin 300072, China; 2 Tianjin Key Laboratory of Neurotrauma Repair, Pingjin Hospital Brain Center, Characteristic Medical Center of PAPF, Tianjin 300162, China; 3 National Engineering Research Center in Biomaterials, College of Biomedical Engineering, Sichuan University, Chengdu 610064, Sichuan, China; 4 Department of Neurosurgery, West China Medical School, West China Hospital, Sichuan University, Chengdu 610041, Sichuan, China; 5 Department of Hematology, The First Affiliated Hospital of Chengdu Medical College, Chengdu 610500, Sichuan, China

doi: 10.1093/rb/rbab047, *Regenerative Biomaterials* 2021;1–20

In the originally published version of this manuscript, the supplementary figures were omitted. The supplementary figures are now available online.

**Figure:** Fig. S1. Schematic illustration of the experimental procedure.

**Figure:** Fig. S2. In vivo degradation curves of 3D-CC-BDNF scaffolds with five mass ratios.

**Figure:** Fig. S3. The image of DCX (green) immunofluorescence staining. Immature neurons were identified by immunostaining with anti-DCX antibody (green) (A) at 7 days after co-culture. Dapi antibody (blue) was performed to counter-stain cell nuclei (A) at 7 days after co-culture. Quantification of the DCX+ cell density (B) and the ratio of DCX+/Dapi (C). Data were presented as mean ± SD, n = 5. *P < 0.05 vs 3D-CC+BDNF. ##P < 0.01 vs 3D-CC. Scale bars = 50 µm in panels (A).

**Figure:** Fig. S4. Biocompatibility testing of scaffolds and human umbilical cord mesenchymal stem cells in vitro. (A) Typical cell morphology of the fourth generation HUCMSCs under a phase contrast microscopy. (B) Expression of CD90, CD105, CD73 and negative molecules (Neg PE) (CD45, CD116, CD19 and HLA-DR). (C-D) Double immunostaining with anti-CD90 antibody (green) and anti-CD105 antibody (red) to identify HUCMSCs. D was an amplified image of the yellow box in C. (E-F) Morphological observation of HUCMSCs cultured on 3D-CC+BDNF (E) and 3D-CC-BDNF (F) under a phase contrast microscope. The image on the right was an amplified image of the yellow box in the image on the left. (G-H) Scanning electron microscopy observation of HUCMSCs growth status in 3D-CC+BDNF (G) and 3D-CC-BDNF (H). (I-J) Double immunostaining with an anti-CD90 antibody (green) and an anti-CD105 antibody (red) to identify HUCMSCs in the 3D-CC+BDNF (I) and 3D-CC-BDNF (J). (K-L) HE staining observation of 3D-CC+BDNF (K) and 3D-CC-BDNF (L) co-cultured with HUCMSCs. (M) Light absorbance of HUCMSCs in the 3D-CC+BDNF and 3D-CC-BDNF co-cultured with HUCMSCs at 1, 3, 5 and 7 days after seeding HUCMSCs. Data were presented as mean ± SD, n = 5. *P < 0.05, **P < 0.01 vs 3D-CC+BDNF. Scale bars = 50 µm in panels (A, C, D, E, F, G, H, K, L), 300 µm in panels (I, J).

